# Perspectives and challenges in patient stratification in Alzheimer’s disease

**DOI:** 10.1186/s13195-022-01055-y

**Published:** 2022-08-13

**Authors:** Carla Abdelnour, Federica Agosta, Marco Bozzali, Bertrand Fougère, Atsushi Iwata, Ramin Nilforooshan, Leonel T. Takada, Félix Viñuela, Martin Traber

**Affiliations:** 1 Universidad Autónoma de Barcelona, Barcelona, Spain; 2 Unit of Neurology and Neuroimaging Research Unit, IRCCS San Raffaele Scientific Institute, and Vita-Salute San Raffaele University, Milan, Italy; 3 Department of Neuroscience “Rita Levi Montalcini”, University of Turin, Turin, Italy; 4 Department of Geriatric Medicine, Tours University Hospital, Tours, France; 5 Department of Neurology, Tokyo Metropolitan Geriatric Hospital, Tokyo, Japan; 6 Abraham Cowley Unit, Surrey and Borders Partnership NHS Foundation Trust, Chertsey, UK; 7 University of Surrey, Guildford, UK; 8 UK Dementia Research Institute – Care Research and Technology Centre, London, UK; 9 Cognitive and Behavioral Neurology Unit, Department of Neurology, University of São Paulo, São Paulo, Brazil; 10 Instituto Neurológico Andaluz, Hospital Victoria Eugenia/Unidad Deterioro Cognitivo, Hospital Virgen Macarena/Faculty of Medicine, University of Seville, Seville, Spain; 11 Product Development Medical Affairs, F. Hoffmann-La Roche Ltd., Basel, Switzerland

**Keywords:** Alzheimer’s disease, Patient stratification, Biomarkers, Mild cognitive impairment, Precision medicine, Early diagnosis, Disease-modifying therapy, Dementia, Stigma, Cultural perceptions of dementia

## Abstract

**Background:**

Patient stratification is the division of a patient population into distinct subgroups based on the presence or absence of particular disease characteristics. As patient stratification can be used to account for the underlying pathology of a disease, it can help physicians to tailor therapeutic interventions to individuals and optimize their care management and treatment regime. Alzheimer’s disease, the most common form of dementia, is a heterogeneous disease and its management benefits from patient stratification in clinical trials, and the development of personalized care and treatment strategies for people living with the disease.

**Main body:**

In this review, we discuss the importance of the stratification of people living with Alzheimer’s disease, the challenges associated with early diagnosis and patient stratification, and the evolution of patient stratification once disease-modifying therapies become widely available.

**Conclusion:**

Patient stratification plays an important role in drug development in clinical trials and may play an even larger role in clinical practice. A timely diagnosis and stratification of people living with Alzheimer’s disease is paramount in determining people who are at risk of progressing from mild cognitive impairment to Alzheimer’s dementia. There are key issues associated with stratifying patients which include the heterogeneity and complex neurobiology behind Alzheimer’s disease, our inadequately prepared healthcare systems, and the cultural perceptions of Alzheimer’s disease. Stratifying people living with Alzheimer’s disease may be the key in establishing precision and personalized medicine in the field, optimizing disease prevention and pharmaceutical treatment to slow or stop cognitive decline, while minimizing adverse effects.

## Background

Dementia is a growing public health concern which affects over 50 million people globally, a total which is projected to grow to more than 150 million by 2050 [1]. Alzheimer’s disease (AD) is the most common type of dementia, predominantly affecting those 65 years and older, and is characterized by a global decline in cognition and an inability to perform daily activities [2]. AD is characterized by the accumulation of amyloid-beta (Aβ) and hyperphosphorylated tau protein that forms senile plaques and neurofibrillary tangles (NFTs) in the brain, respectively [2]. These neurotoxic proteins play a role in neuronal cell death which correlates with the clinical manifestation of AD [2]. The clinical features of AD and other causes of dementia often overlap and include symptoms such as memory decline, apathy, anxiety, and depression, which often occur in earlier stages [2]. Later symptoms include impaired communication and speech, disorientation, and confusion [2].

There are two classes of medications approved by the US Food and Drug Administration (FDA) for the symptomatic treatment of AD: cholinesterase inhibitors (donepezil, galantamine, and rivastigmine), and N-Methyl-D-aspartate receptor antagonists (memantine); both classes may improve symptoms but do not intercept disease pathology [2]. In June 2021, the first disease-modifying therapy (DMT), aducanumab, was approved by the FDA using the accelerated approval pathway for the treatment of AD [3, 4]. Aducanumab is the first approved treatment directed at the underlying pathology of AD, namely, the presence of Aβ in the brain [3]. While efficacy studies of aducanumab were conducted in those with prodromal-to-mild (early) AD, there are currently few guidelines publicly available to identify who may benefit most from treatment [4]. Identifying and appropriately selecting patients for DMTs (such as aducanumab) by accurately characterizing them is of great importance, as several agents are in late-phase clinical development, including donanemab, gantenerumab, and lecanemab [5]. Patient stratification may be harnessed as a tool to identify individuals properly to optimize treatment outcomes in terms of benefits and risks from current or future treatment options [6].

Across the field of healthcare, there is a growing need to provide more effective and safer care that is tailored to the individual person with a disease [7]. Yet, many complex diseases, such as AD, are heterogeneous in their clinical presentation, severity, and response to therapies [8–10]. This makes it difficult to determine the appropriate clinical course of action, as people may exhibit multiple phenotypes on distinct disease trajectories [8, 9]. Patient stratification is an approach that aims to cluster people with a disease into more homogeneous groups by classifying them according to factors considered highly important to the disease [10], so that interventions can be offered to those who will benefit the most. Proposed patient stratification schemes in AD have focused on those that can account for differing contributions from all relevant risk factors, such as genetic predisposition, molecular indicators, and pathologic stage disease [10]. A patient stratification scheme may account for molecular indicators, pathologic staging, and relevant risk factors including genetic predisposition [10]. Official bodies such as the World Health Organization have recognized that the stratification of the health risks of people with chronic diseases could strengthen population health management and enable the provision of better-tailored services [11]. It can also ensure individual needs are met in a timely and efficient manner [12]. The documented benefits of patient stratification in advancing care in other disease areas highlight its potential for AD. Oncology is probably the highest-profile field where patient stratification has made its impact. For example, the stratification of breast cancers into integrative clusters has been associated with distinct clinical courses and response to therapy [13]. Consequently, breast cancer mortality has decreased by 40% over the past three decades [14]. Elsewhere in oncology, immunotherapies targeting immune checkpoints have led to exciting new therapeutic strategies, but there is still a need to understand which patient groups would benefit most from the treatments. Since interactions between tumor and immune cells in the tumor microenvironment influence the effectiveness of immunotherapy, more detailed understanding of the tumor microenvironment should enable better patient stratification to improve clinical outcomes [15]. Patient stratification can revolutionize disease treatment by tailoring therapeutic options to the individual, as seen in the molecular targeting of tumor biomarkers in breast cancer [14]. There are numerous barriers to patient stratification in AD, and a paucity of literature discussing patient stratification in everyday AD clinical practice. In this review, we discuss the importance, challenges, and evolution of patient stratification in AD.

## Potential for patient stratification in AD

### AD diagnosis: stratifying AD from other dementias

AD is a multifactorial disease, and the first step to stratification of AD is accurate differential diagnosis of AD from other causes of dementia [2]. The symptoms and biomarker abnormalities observed in AD may overlap with other dementias, resulting in misdiagnosis [2, 16]. Criteria developed over the past 15 years from both the US National Institute on Aging and the Alzheimer’s Association (NIA-AA) and the International Working Group for New Research Criteria for the Diagnosis of AD (IWG) require biomarker evidence of disease pathology for differential diagnosis of AD [16]. The 2018 NIA-AA research criteria introduced a classification scheme diagnosing AD biologically based on the presence of Aβ, pathologic tau, and neurodegeneration/neuronal injury (AT[N] framework) [17]. The 2021 IWG perspective recommends a clinical–biological diagnosis of AD, which is restricted to those who have specific AD clinical phenotypes that are then confirmed by biomarker evidence of both Aβ and tau [16]. A cognitively unimpaired person with biomarker evidence of both amyloid and tau would be diagnosed with “preclinical AD” according to the NIA-AA research criteria, but be diagnosed as “at risk for progression to prodromal AD or AD dementia” under the new IWG guidelines [16, 17].

### Identifying people at risk for AD clinical progression

The phenotypic and genotypic variability in AD makes early diagnosis and stratification of patients, at the prodromal or an earlier stage of AD, challenging [18]. This difficulty stems from variable disease trajectories and complex neurobiology [18]. People with AD are generally staged according to the severity of clinical symptoms as cognitively unimpaired, having mild cognitive impairment (MCI) due to AD (also called prodromal AD), or AD dementia [2, 17]. AD dementia is further classified as mild, moderate, or severe [17]. There can be a long pre-symptomatic period in AD, with pathology accumulating for up to 20 years prior to the onset of symptoms [2]. Although the IWG recommendations are not widely used in clinical settings, the IWG recommends against AD biomarker assessment in cognitively unimpaired people, a recommendation based, in part, on cross-sectional evidence observing AD brain lesions in cognitively unimpaired people in both neuroimaging and post-mortem examinations [16]. As not everyone with biomarker evidence of AD will progress [16], it is imperative to identify those who are at risk for disease progression. Additionally, people with AD progress along the continuum at widely varying rates, and research is ongoing to identify specific factors that influence the rate of progression in AD [19]. Identifying individuals at an increased risk of progression aims to ensure that those in need of urgent treatment are given access [20]. Those who are at a higher risk of progression may be appropriate candidates for more aggressive pharmacologic therapy, namely DMTs [20]. For people at lower risk of progression, active monitoring, lifestyle interventions, and symptomatic therapies may be more appropriate [2]. Evidence-based AD risk reduction is feasible in clinical practice, tailoring interventions through a mix of neuropsychological, clinical, and laboratory assessments [21].

A breadth of research has been conducted to identify prognostic factors for progression to MCI and dementia, as detailed in various meta-analyses and systematic reviews [22–24]. As expected, one of the first indicators of AD risk is personal memory complaints; those with subjective cognitive decline are at twice the risk of developing dementia compared with people without subjective memory complaints [23]. Anxiety is also associated with an increased risk of progression from normal cognition to MCI or dementia [24]. Once MCI due to AD is detected, the risk of progression to AD dementia is increased at older ages, in women, in those with low-level education, in people with at least one copy of the apolipoprotein E ε4 (*APOE ε4*) allele, in those with impaired cognition, and/or in those with comorbidities such as depression, diabetes, or hypertension [18, 22, 25, 26]. Progression from MCI due to AD to AD dementia is also linked with biomarkers of AD pathology such as cerebrospinal fluid (CSF) markers of phosphorylated tau (pTau) and total tau (tTau)/Aβ(1–42) ratio, with brain atrophy (hippocampal, medial temporal lobe, entorhinal) and parieto-temporal hypometabolism on [^18^F]-fluorodeoxyglucose (FDG) positron emission tomography (PET) [22, 27]. In addition to the risk of progression, the speed of progression is also impacted by age, gender, and *APOE ε4* genotype [28].

A variety of modifiable risk factors are also associated with progression. Modeling indicates that up to 40% of dementia could be prevented or delayed by intervening in risk factors that can be modified during various stages of life [29]. Elimination of 12 potentially modifiable risk factors may help reduce dementia prevalence. In early life (<45 years), a 7% reduction in dementia prevalence was suggested if low education level as a risk factor was eliminated. In midlife (age 45–65 years), the risk factors would be hearing impairment (8% risk), traumatic brain injury (3%), hypertension (2%), alcohol consumption of >21 units per week (1%), and obesity (1%). In later life (age >65 years), the risk factors would be smoking (5% risk), depression (4%), social isolation (4%), physical inactivity (2%), exposure to air pollution (2%), and diabetes (1%) [29]. Modifiable and non-modifiable risk factors of AD modulate many aspects of the disease course, including onset, clinical manifestations, and prognosis [30].

### Patient stratification in clinical practice

**Fig. 1:**
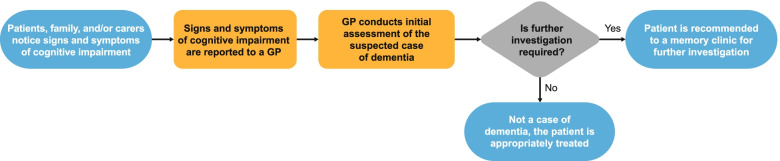
Initial diagnostic pathway by GPs. GP, general practitioner

While literature regarding stratification for treatment with a DMT is theoretical [20, 31], there are conclusions which may be drawn based on existing treatment paradigms and gaps in AD clinical practice. In some countries, such as the UK, general practitioners (GPs) play a major role in the early stages of patient identification and stratification (Fig. 1) [32].

Outside of a clinical trial setting, non-specialists, such as GPs, are typically the first to screen for and evaluate patients with MCI or dementia. Patients, family, or caregivers who suspect symptoms of dementia often report these symptoms at primary care clinics [32]. These symptoms include impaired memory, mood and personality changes, and psychological symptoms such as depression and anxiety, all of which could be caused by AD [32]. Subsequently, GPs conduct initial brief assessments (e.g., patient history regarding psychological and behavioral symptoms, physical examination, relevant blood and urine tests, and cognitive tests using a validated instrument) to support the suspicion of cognitive decline and rule out any other potential causes of these symptoms. Patients are either referred to memory clinics for further dementia assessment or treated as non-dementia cases [32]. GPs are essentially stratifying patients into three categories: those with suspected dementia, those with cognitive problems without dementia, and those who are not cognitively impaired but still have complaints. Memory clinics conduct further investigations to diagnose patients, taking into consideration the initial assessments from GPs (Fig. 1). These additional investigations include in-depth cognitive and behavioral assessments, structural neuroimaging (brain computerized tomography [CT] or magnetic resonance imaging [MRI]) scans, PET scans, genotyping, and fluid biomarker analysis (discussed in more depth in the next section) [10]. Brain CT or MRI is an integral part of the diagnostic pathway in patients with suspected AD dementia; however, other methods such as PET scans, APOE genotyping, and the analysis of the core AD CSF biomarkers are not routinely used in clinical settings [33–36]. Diagnostic tests conducted by AD specialists can be used to stratify patients further, which can help inform the treatment and care pathway. In terms of DMTs, this could mean selecting patients who will most likely benefit from treatment.

The dementia detection rate differs between countries, especially between higher- and lower-income countries, and depends on a multitude of factors [6, 37]. These factors are mainly based around how developed the healthcare system is, training of GPs and specialists, and cultural perceptions of dementia [37]. In the UK (where a quarter of the population will be over 65 years by 2050), only two-thirds of people with dementia receive a formal diagnosis, while in Brazil (where the population age 65 and older is projected to triple by 2050) the proportion is even lower, with one-quarter of people with dementia receiving a formal diagnosis [37–39]. This highlights the heterogeneity regarding diagnostic processes in different countries. A factor in the staggering disparity between the dementia diagnosis rates in the UK and Brazil is the inadequate availability of memory clinics in Brazil, which are often limited to universities; this can cause long waiting lists for those who live in remote areas, and can drastically increase the time it takes to reach a diagnosis of dementia [37]. Additionally, neuroimaging facilities, particularly MRI, are scarce [37]. Secondary care aside, GPs often have very limited time to conduct a cognitive screening test (less than 10 minutes), which is similar between the UK and Brazil, lack the confidence to diagnose dementia, and are often unaware of guidelines and protocols for evaluating and managing patients with memory complaints [37]. Cognitive decline is often viewed as a normal part of aging among patients and their families in Brazil [37]. Words such as “Alzheimer’s” or “dementia” have a stigma associated with them which is worse among high-income families who frequently feel ashamed and consequently hide the condition from others [37].

### Patient stratification to enrich clinical trial populations

A challenge for clinical trials is identifying people with AD who are likely to progress rapidly, as they are more likely to indicate whether a new drug is efficacious over the duration of a late phase clinical trial [40]. Historically, there have been highly variable trajectories of cognitive decline in the placebo group of randomized clinical trials in AD, highlighting the importance of careful selection of clinical trial inclusion and exclusion criteria [41]. Stratifying patients by those who may likely benefit from a DMT, according to current clinical thinking, will be crucial for understanding the prognosis for defined patient subgroups [21]. In recent years, investigation of DMTs has focused on slowing disease progression and targeting early (i.e., prodromal-to-mild) AD [5]; accordingly, patients are stratified for earlier stages of disease [10]. The Free and Cued Selective Reminding Test (FCSRT) is a neuropsychological test used to evaluate episodic memory and can be used to enrich a clinical trial population [40, 42]. The FCSRT has been incorporated into the inclusion criteria of Roche-sponsored pivotal trials such as CREAD, a phase III trial of crenezumab, and GRADUATE, a phase III trial of gantenerumab to help identify participants with an elevated risk of developing AD dementia [40, 43]. Including the FCSRT as part of the eligibility criteria increases the potential of enriching a clinical trial population with individuals with early AD who are likely to progress during the study [40]. This is essential in investigating DMTs targeting the earlier stages of AD, as the ability of a DMT to demonstrate efficacy versus placebo is based partly on the rate of decline, or progression, observed within the placebo group. The trajectory of the placebo group helps to determine the treatment difference at the end of a clinical trial [40]. TRAILBLAZER-ALZ, a phase II trial of donanemab in participants with early symptomatic AD, is another recent example of the use of patient stratification to enrich a clinical trial population. In addition to conventional eligibility criteria, participants were required to have flortaucipir PET scans with evidence of pathologic tau deposition but with quantitative tau levels below an upper threshold. This resulted in a decrease in the variable trajectories of clinical decline [44]. Although flortaucipir PET scans have demonstrated their capability as an enrichment tool, PET scans are considered both invasive and expensive and therefore are not utilized in routine diagnosis [35, 45]. An important aspect of stratifying participants for global clinical trials is the considerable regional heterogeneity in terms of patient characteristics and outcomes, and therefore of symptom manifestation and progression [46].

### Identifying people at risk for amyloid-related imaging abnormalities

There is a pressing need to utilize safety biomarkers, so as to enable the early detection or avoidance of amyloid-related imaging abnormalities (ARIA) [47]. ARIA is a common side effect of anti-Aβ-targeting monoclonal antibodies. While ARIA is mostly asymptomatic, it can manifest clinically in headaches, confusion, and neuropsychiatric symptoms [47]. Individuals may also experience mildly asymptomatic ARIA which quickly resolve following the discontinuation or dose modification of anti-Aβ therapy [47]. ARIA are divided into two classes: ARIA—edema (ARIA-E) is a vasogenic edema determined by MRI (i.e., sulcal effusion on fluid-attenuated inversion recovery images), which indicates inflammation of the affected vessels [47]. ARIA—hemorrhage (ARIA-H) is again detected by MRI and characterized by a signal of hemosiderin deposits involving microhemorrhages and superficial siderosis on T2*-weighted gradient echo or susceptibility-weighted imaging, indicating cerebral amyloid angiopathy [47]. As it is difficult to predict the occurrence of ARIA, current FDA guidelines for enrolling participants in clinical trials assessing DMTs recommend MRI evaluations to exclude participants with ≥5 microhemorrhages and with any evidence of superficial siderosis or prior parenchymal hemorrhage [47]. The mechanisms which lead to the development of ARIA are not fully understood; however, it has been well demonstrated that high doses of anti-Aβ therapy and *APOE* ε4 carriers are at a higher risk of developing ARIA [47]. In EMERGE and ENGAGE, two phase III randomized clinical trials of aducanumab in participants with MCI due to AD or mild AD dementia, the most common adverse event in those receiving a high dose of aducanumab was ARIA-E. This is consistent with prior aducanumab studies and safety data from other anti-Aβ therapies. ARIA-E incidence was also higher in *APOE* ε4 carriers than *APOE* ε4 noncarriers, and was found to be dose-dependent when assessed by *APOE* carrier status [48]. Developing a reliable method to stratify participants based on the risk of developing ARIA may mitigate the occurrences of ARIA, a trend which may also translate well into clinical practice [47].

### Future identification of eligibility for a disease-modifying therapy

It is expected that, as DMTs become more widely available, the demand for an early and timely AD diagnosis and treatment will increase [20]. As a growing number of DMTs in development target amyloid or tau pathology [5], individuals without biomarker-confirmed evidence of these pathologies may not be suitable for an anti-Aβ or anti-tau DMT. Additionally, those with biomarker evidence of AD pathology but normal cognition may not progress to AD dementia [16] and may not be candidates for a DMT. Furthermore, people with more advanced AD may not be suitable for the majority of the DMTs in the AD pipeline. These putative DMTs mainly target disease onset or disease progression in the earlier stages of AD. A robust patient stratification scheme will work to exclude people who are ineligible for DMTs based on a combination of cognitive and functional assessments, genetic risk factors, demographic and lifestyle factors, and AD pathology [10].

## Methods and diagnostics for AD stratification

**Fig. 2:**
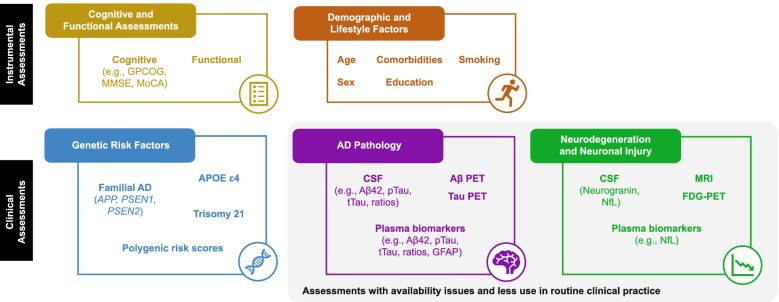
Tools for AD stratification. Aβ, amyloid-β; *APOE* ε4, apolipoprotein E ε4; *APP*, amyloid precursor protein; CSF, cerebrospinal fluid; FDG, fluorodeoxyglucose; GFAP, glial fibrillary acidic protein; GPCOG, General Practitioner Assessment of Cognition; MMSE, Mini-Mental State Examination; MoCA, Montreal Cognitive Assessment; MRI, magnetic resonance imaging; NfL, neurofilament light chain; PET, positron emission tomography; *PSEN*, presenilin; pTau, phosphorylated tau; tTau, total tau

Cognitive and functional assessments, genotyping, CSF and PET imaging biomarkers, and MRI are some of the current methods which can be used to stratify patients according to their AD pathology and clinical stage (Fig. 2). These methods of stratifying can provide results that are both “dynamic” or “static” in nature, either evolving as AD progresses or remaining the same throughout (Table 1). This should be taken into consideration when using these methods to stratify patients.

**Table 1:** Static and dynamic stratification factors

Static stratification factors	Dynamic stratification factors
Genotyping	Cognitive and functional assessments
*APOE* status	CSF biomarkers
Sex	PET imaging biomarkers
	MRI

*APOE* apolipoprotein E, *CSF* cerebrospinal fluid, *MRI* magnetic resonance imaging, *PET* positron emission tomography

Cognitive assessment is traditionally the first step to a diagnosis of AD or other dementias [16]. Assessment scales have been developed that evaluate cognition, function, and behavior for both clinical practice and research [32, 49]. Cognitive screening tools are quick, economical, and non-invasive [49]. These evaluation scales range from brief assessments used to screen for dementia to more detailed tools assessing different cognitive domains [49]. No singular cognitive assessment tool is recognized as the best assessment tool across the AD continuum; some tools are optimized for specific stages of dementia. The Mini-Mental State Examination (MMSE) is the most commonly used screening instrument for detection of cognitive impairments associated with AD and only takes 5 to 10 min to administer [49]. However, the MMSE may have limited discrimination between the cognitively unimpaired and those with MCI, as well as between AD and other dementias [49]. The General Practitioner Assessment of Cognition (GPCOG) is a two-part, 4- to 6-min test, freely available online to screen for dementia in primary care which has been recognized as an effective screening tool and a viable alternative to the MMSE [50]. The Montreal Cognitive Assessment (MoCA) is a 10- to 15-min test that is validated for MCI in a population-based cohort as well as for MCI and dementia in memory clinics [49]. Assessing episodic memory is especially helpful for differential diagnosis of AD dementia, as this domain is one of the first to decline in typical forms of AD but not in other dementias [2, 49]. The preclinical Alzheimer’s cognitive composite (PACC) measures episodic memory, executive function, and global cognition [51]. PACC is currently used in secondary prevention trials as it can capture decline in amyloid-positive, clinically normal, older adults [51]. The addition of category fluency, a semantic category, to PACC (PACC5) adds unique information about amyloid Aβ-related decline [51]. Despite the proven utility of these screening tools, studies have demonstrated that sociodemographic variables, such as age and education level, and health variables, such as medical history, have implications for the performance of cognitive screening tests [52].

As previously discussed, there are a variety of demographic, genetic, and environmental risk factors that are associated with modulation of cognitive decline which are key components for stratifying for risk of AD and AD progression [2, 16, 22, 29]. Personal history and demographic factors that have been linked to increased AD risk include older age, female sex, low education level, smoking, excessive alcohol consumption, low physical activity, low social contact, and exposure to air pollution [16, 29]. Medical history also impacts AD risk, which is increased by frailty, depression, hearing impairment, traumatic brain injury, obesity, diabetes, and hypertension [2, 16, 22, 29]. Genetic mutations in amyloid precursor protein (*APP*), presenilin (*PSEN*)*1*, and *PSEN2* lead to accumulation of Aβ which results in Aβ deposition and aggregation in the brain; these are the driving mutations for familial AD [53]. The *APOE* ε4 genotype is a major risk factor for the development of AD, and the risk increases further from 47% in heterozygous *APOE* ε4 carriers to 91% in homozygous *APOE* ε4 carriers, regardless of ethnicity [2, 18, 54]. Additionally, in clinical trials of amyloid-targeted antibodies as DMTs for AD, *APOE* ε4 has been linked to an increased risk for ARIA [54].

The characteristic pathology of AD, Aβ plaques and tau NFTs, may be detected using PET scans with targeted ligands and CSF examination [17, 55, 56]. Three amyloid PET imaging agents ([^18^F]-florbetapir, [^18^F]-florbetaben, and [^18^F]-flutemetamol) and one tau PET ligand ([^18^F]-flortaucipir) are approved by the FDA for medical use and commonly applied in clinical trials of AD [56, 57]. PET scans may be quantified using standardized uptake value ratios to determine whether a person is above or below the amyloid positivity threshold associated with AD [58]. AD pathology can also be detected using validated CSF biomarkers for AD [16, 59]. Key CSF biomarkers for AD, including decreased Aβ(1–42), increased tTau, and increased pTau, are measured with commercially available enzyme-linked immunosorbent assay and electrochemiluminescence immunoassay kits [59, 60]. PET or CSF confirmation of AD pathology allows for definitive diagnosis of AD according to either the IWG or NIA-AA criteria [16, 17], which encourages informed decision-making in AD treatment and care management [21]. Longitudinal PET or CSF measures may also be useful to monitor disease progression. The Roche Elecsys® AD CSF immunoassays are an example of a diagnostic tool that can be used to detect AD pathology. Elecsys® pTau/Aβ(1–42) and tTau/Aβ(1–42) are robust biomarkers for predicting the risk of clinical decline and conversion to dementia in non-demented patients [61]. One exciting development in fluid-based biomarker diagnostics is the NeuroToolKit (Roche Diagnostics International Ltd.), a panel of 12 CSF biomarker assays covering Aβ pathology, neurodegeneration, neurofilament light chain (NfL), and α-synuclein metabolism. Investigation is ongoing to evaluate these biomarkers as a tool for selecting patient populations and as pharmacodynamic biomarkers [62]. Another contribution to improving fluid-based diagnostic approaches in AD has been the commercial development of liquid chromatography tandem mass spectrometry-based assays that simultaneously quantify Aβ isoforms in human plasma and determine common *APOE* prototype [63]. According to the company behind this approach, in a study of 686 participants over the age of 60 with cognitive impairments or dementia, a sensitivity of 92% and a specificity of 76% were achieved when compared with amyloid plaque deposits confirmed with a PET scan [64]. Another reported study examined the diagnostic accuracy of plasma pTau217 for AD [65]. In a cross-sectional study of 1402 participants from three selected AD cohorts, plasma pTau217 discriminated AD from other neurodegenerative diseases with performance that was deemed significantly better than established AD plasma- and MRI-based biomarkers, but not significantly different from key CSF- or PET-based biomarkers [65]. While such research is promising, the authors highlighted that further research would be needed to validate the findings in unselected and more diverse populations [65]. Blood-based biomarkers for AD pathology are also being investigated, in the hope of validating them as a more economical and less invasive option [66]. However, blood-based biomarkers are not yet widely available for AD diagnosis or stratification [66]. The latest advancement in blood-based biomarkers is the *QPLEX*™ *Alz plus assay kit* [67]. Preliminary evidence suggests that this assay kit can be used to predict cerebral amyloid deposition using blood-based biomarkers, but independent validation is still required [67]. Should blood-based biomarkers become available, they may alleviate the bottleneck in stratifying patients for treatment with a DMT [66, 68]. Biomarkers of neurodegeneration, while not specific to AD, may also be useful for stratification [17]. Brain MRI can be used to exclude other causes of cognitive impairment and detect atrophy in different regions of the brain, and correlates closely with other symptoms of AD [17, 22]. Some CSF biomarkers are indicative of neurodegeneration or other neural damage, including NfL, which is elevated in many neurological conditions, and neurogranin, which may be differentially elevated in those with AD [69, 70]. FDG PET often shows reduced cerebral glucose metabolism in people with AD compared with healthy individuals [27]. Although this is not fully specific to AD, the pattern of FDG PET hypometabolism varies in different dementias and often contributes to the differential diagnosis [27]. These biomarkers help characterize downstream effects of AD pathology at the individual patient level.

## Major challenges to stratification in AD

A well-defined patient stratification scheme will be crucial to identify patients who will likely benefit from a DMT. There are many challenges and counterarguments that pertain to the initial diagnosis of AD due to cultural stigma or issues with GP preparedness, as well as healthcare system readiness and availability of biomarker testing.

### Limitations of a biomarker-based diagnosis of Alzheimer’s disease

A major challenge in clinical practice is to distinguish AD from other neurodegenerative disorders and the presence of comorbidities. The in vivo presence of biomarkers of AD lesions could warrant AD as a primary diagnosis; however, these lesions can be found in other neurodegenerative disorders such as dementia with Lewy bodies [16]. Similarly, cases with pure AD pathology are only found in 3–30% of neuropathologic series of people with AD dementia at post-mortem examination [16]. Biomarkers for the pathologic changes that underlie other neurodegenerative diseases often found in people with dementia are currently under investigation, and as a result, distinguishing AD from other neurodegenerative diseases mainly relies on clinical phenotype or post-mortem examination [16].

### Cultural perceptions and understanding of Alzheimer’s disease

A timely AD diagnosis allows for earlier intervention; however, the stigma of a diagnosis in the absence of effective therapeutic options may prevent individuals from seeking help [6, 71]. The social stigma of an AD diagnosis is palpable in some societies and cultures, which may further discourage patients from reporting early symptoms to their GPs and delay early intervention or support [37, 71]. Particularly after the first few years after a diagnosis of dementia, psychological complications may contribute to an increased risk of suicide [6]. Another major issue is the lack of awareness from patients, their families, and GPs about dementia and its symptoms [6]. Patients have reported dismissing symptoms of dementia as a normal part of aging, and therefore do not disclose them to GPs [71]. Some GPs have also reported that they struggle to differentiate cognitive impairment from normal aging [71]. The normalization of symptoms causes delays in seeking help [6]. Patients and GPs often prioritize physical over mental health, which may cause them to be dismissive to symptoms of dementia [6]. Aside from a diagnosis of dementia directly affecting the patient, families and caregivers around the patient are also negatively affected. They are often reluctant to take on the caregiving burden for a variety of reasons. This may be a result of fear and anxiety, time constraints, and/or changes to the family dynamic after a diagnosis [6].

To mitigate the impact of cultural perceptions and the lack of understanding of AD on diagnosis rates, increased efforts with educational campaigns around dementia, its symptoms, and the importance of an early diagnosis are essential. A better understanding of dementia by patients, families, and caregivers will encourage patients to actively seek a formal diagnosis and develop a disease management plan. Promotion of support services to manage AD may also alleviate the burden on both patients and families [71].

### Improving collaboration between primary care and memory clinics

A current challenge is enabling GPs better to identify patients who would benefit from further assessment and treatment at a memory clinic, as some GPs admit having difficulty identifying early symptoms of AD and/or lack confidence in their ability to accurately diagnose AD [6]. Healthcare systems are not prepared for the extensive screening required to assess for MCI. It was estimated that over 80 million patients in 2019, across France, Germany, Italy, Spain, Sweden, and the UK, would undergo cognitive screening for MCI due to AD and that over 10 million would screen positive [31]. These numbers may increase as DMTs become available. The number of specialists available in France, the UK, and Spain is a significant rate-limiting factor which will exacerbate waiting times across the diagnostic pathway [6, 31]. A contributing challenge is the poor coordination of the diagnostic process between primary care clinics and memory clinics [6]. In the future, GPs will need support in the difficult task of explaining that DMTs may not be suitable for patients with advanced disease.

Collaborative care models with multidisciplinary teams are a viable solution to improving the collaboration between primary care and memory clinics. Over the past two decades, there has been increasing use of collaborative care approaches to developing new models of care for older patients, leading to improved quality, efficiency, and outcomes of care. These models require the implementation of a robust dementia care strategy, training of a healthcare workforce from different disciplines, partnerships with healthcare delivery systems, and agreements with third-party payers to recognize the cost savings to support these changes in specialty practice [72].

### Biomarkers and healthcare system readiness

The potential requirement of widespread diagnostic testing for the rollout of DMTs will be a resource-intensive and an economically demanding burden on healthcare systems [20, 31, 68]. Brain MRI may be used in the diagnostic process to assist in the differential diagnosis of other causes of cognitive impairment, and to detect atrophy in different regions of the brain [17, 22]. Both PET imaging and CSF assessments are diagnostic methods that are only accessible in specialized centers. PET imaging may be cost-prohibitive, and CSF testing may be perceived to be invasive; owing to these challenges, these methods are not utilized in routine diagnosis [45]. A sudden and sustained increase in demand for biomarker-confirmed diagnosis by AD specialists could strain memory clinic resources [20, 31, 68]. While healthcare systems may face challenges in offering biomarker-confirmed diagnoses, the potential advances in dealing with AD in clinical care must not be ignored. For example, biomarker profiles could allow prediction of personalized trajectories of future cognitive progression in patients. A recent biomarker-based prognostic model was applied to data from a large cohort of patients [73]. Featuring three CSF and two MRI measures, the model significantly improved personalized prognosis when incorporating biomarker information on top of cognition and demographic data [73]. The authors concluded that biomarkers that are being routinely collected in some clinical settings seem to have an unused potential to improve patient prognosis of future cognitive decline in clinical practice and may also be useful for optimizing clinical trial design [73]. There is also hope that blood-based AD biomarkers may counteract the anticipated diagnostic capacity limitations in some healthcare systems by providing testing at lower cost and higher accessibility [74]. Plasma measurements of pTau217 and NfL, combined with basic demographics, significantly predicted both change in cognition and subsequent AD dementia in 435 cognitively unimpaired elderly individuals [74]. It has been predicted that a combination of pTau and other accessible biomarkers might provide accurate prediction about the risk of developing dementia, and promising results have been achieved using the BioFINDER and Alzheimer’s Disease Neuroimaging Initiative patient cohorts [75]. Nevertheless, the application of these biomarker-based approaches to routine healthcare is some way off at present [75]. In particular, further validation of results is required in large, unselected, and ethnically diverse primary care populations with a lower pre-test probability of underlying AD [75]. The validation of a diagnostic method that is capable of being administered in a resource-limited environment, such a blood-based testing kit, would be beneficial in relieving the stress on specialty centers [68]. Stratifying people with AD by their susceptibility to adverse events of special interest, such as ARIA, will impose an additional burden on memory clinic resources. Subsequent monitoring of ARIA is also required to ensure patient safety [76]. Stratification based on the *APOE* ε4 genotype, a major risk factor for the development of AD, will be required to determine the likelihood of dementia, disease progression, and possible risk for ARIA [28, 54].

## The potential for personalized AD treatment and care

Stratification may play a critical role in decreasing the impact of an AD diagnosis on families [77]. Family caregivers often report work-related stress that stems from having to adjust their work schedule around patient needs, with some caregivers having to give up their own jobs entirely [77]. Caring for people with dementia is a heavy financial burden, and many families experience challenges balancing the time needed to care for the patient and time for other activities [77]. The importance of stratification, personalized treatment strategies, and ultimately precision medicine in AD care may increase rapidly with the approval of aducanumab and other potential DMTs in phase III development [5, 76]. The goal of precision medicine is to optimize disease prevention and pharmaceutical treatment to slow or stop clinical decline, while minimizing adverse effects [21]. Precision medicine takes into consideration an individual’s specific genotype, phenotype, biomarker profile, and/or psychosocial characteristics to determine optimal treatment and care [21]. Determining a patient’s risk of developing AD and understanding underlying molecular mechanisms behind their pathology are two important factors in applying precision medicine [21].

## Conclusions

Patient stratification is an increasingly important tool in AD treatment and is crucial to determine which patients are at risk of progression to MCI due to AD and AD dementia. A variety of diagnostic techniques are available to stratify patients based on clinical disease characteristics, biomarkers of disease pathology, genotype, and demographic risk factors. However, currently, the heterogeneous nature and complex neurobiology of AD makes stratification of patients difficult. Aside from the complex molecular pathology associated with AD diagnosis, early detection and stratification come with an array of challenges including cultural stigma and physician training. Quickly streamlining the healthcare system for AD diagnosis and stratification is imperative, given the recent approval of a first DMT.

## Data Availability

Not applicable.

## Abbreviations

Aβ: Amyloid-β
AD: Alzheimer’s disease
*APOE* ε4: Apolipoprotein E ε4
ARIA: Amyloid-related imaging abnormalities
ARIA-E: Amyloid-related imaging abnormalities—edema
ARIA-H: Amyloid-related imaging abnormalities—hemorrhage
*APP*: Amyloid precursor protein
CSF: Cerebrospinal fluid
CT: Computerized tomography
DMT: Disease-modifying therapy
FCSRT: Free and Cued Selective Reminding Test
FDA: Food and Drug Administration
FDG: Fluorodeoxyglucose
GP: General practitioner
GPCOG: General Practitioner Assessment of Cognition
IWG: International Working Group for New Research Criteria for the Diagnosis of AD
MCI: Mild cognitive impairment
MMSE: Mini-Mental State Examination
MoCA: Montreal Cognitive Assessment
MRI: Magnetic resonance imaging
NfL: Neurofilament light chain
NFT: Neurofibrillary tangles
NIA-AA: National Institute on Aging and the Alzheimer’s Association
PACC: Preclinical Alzheimer’s cognitive composite
PET: Positron emission tomography
*PSEN*: Presenilin
pTau: Phosphorylated tau
tTau: Total tau
